# Statistical methods leveraging the hierarchical structure of adverse events for signal detection in clinical trials: a scoping review of the methodological literature

**DOI:** 10.1186/s12874-024-02369-1

**Published:** 2024-10-28

**Authors:** Laetitia de Abreu Nunes, Richard Hooper, Patricia McGettigan, Rachel Phillips

**Affiliations:** 1 Wolfson Institute of Population Health, Queen Mary University of London, London, UK; 2 William Harvey Research Institute, Queen Mary University of London, London, UK; 3 Imperial Clinical Trials Unit, School of Public Health, Imperial College London, London, UK

**Keywords:** Clinical trials, Harm, Methodology, Scoping review, Hierarchical models, Bayesian methods, Signal detection, Error control, Multiple testing, False discovery rate

## Abstract

**Background:**

In randomised controlled trials with efficacy-related primary outcomes, adverse events are collected to monitor potential intervention harms. The analysis of adverse event data is challenging, due to the complex nature of the data and the large number of unprespecified outcomes. This is compounded by a lack of guidance on best analysis approaches, resulting in widespread inadequate practices and the use of overly simplistic methods; leading to sub-optimal exploitation of these rich datasets. To address the complexities of adverse events analysis, statistical methods are proposed that leverage existing structures within the data, for instance by considering groupings of adverse events based on biological or clinical relationships.

**Methods:**

We conducted a methodological scoping review of the literature to identify all existing methods using structures within the data to detect signals for adverse reactions in a trial. Embase, MEDLINE, Scopus and Web of Science databases were systematically searched. We reviewed the analysis approaches of each method, extracted methodological characteristics and constructed a narrative summary of the findings.

**Results:**

We identified 18 different methods from 14 sources. These were categorised as either Bayesian approaches (*n*=11), which flagged events based on posterior estimates of treatment effects, or error controlling procedures (*n*=7), which flagged events based on adjusted *p*-values while controlling for some type of error rate. We identified 5 defining methodological characteristics: the type of outcomes considered (e.g. binary outcomes), the nature of the data (e.g. summary data), the timing of the analysis (e.g. final analysis), the restrictions on the events considered (e.g. rare events) and the grouping systems used.

**Conclusions:**

We found a large number of analysis methods that use the group structures of adverse events. Continuous methodological developments in this area highlight the growing awareness that better practices are needed. The use of more adequate analysis methods could help trialists obtain a better picture of the safety-risk profile of an intervention. The results of this review can be used by statisticians to better understand the current methodological landscape and identify suitable methods for data analysis - although further research is needed to determine which methods are best suited and create adequate recommendations.

**Supplementary Information:**

The online version contains supplementary material available at 10.1186/s12874-024-02369-1.

## Background

In randomised controlled trials (RCTs) with efficacy-related primary outcomes, we also collect, analyse and report data on harm outcomes. To monitor harm, we collect *adverse events* (AEs), defined as “any untoward medical occurrence after exposure to a medicine, which is not necessarily caused by that medicine” by the European Medicines Agency [1]. When there is reasonable evidence of a causal link between the medicine and the event, we describe it as an *adverse reaction*. Events which arise throughout a trial but were not prespecified are referred to as *emerging events*; their total number can reach several hundred for a single trial. As a result, trial statisticians can be faced with multiple unprespecified outcomes to analyse.

A hypothesis testing framework is often adopted for between-arms comparisons of these outcomes, but there are significant issues with this approach [2, 3]. Trials are powered based on their primary efficacy outcomes, and therefore lack the statistical power to allow investigators to make definitive conclusions about potential harms. The large number of outcomes to test can lead to inflated rates of false positives if multiplicity is not controlled for. Conversely, commonly used multiplicity adjustment methods such as the Bonferroni correction tend to be too conservative and true signals are more likely to be missed [4, 5].

Thus, analysis of emerging events should be treated as exploratory and conducted under the framework of *signal detection* : the aim is to detect *signals* indicating that some AEs could potentially be adverse reactions and to flag those events for further analysis - without drawing definitive safety conclusions [2].

Reviews of AE collection, analysis and reporting in trials [3, 6, 7] have documented the frequent presence in practice of the aforementioned issues and issues with the choice and reporting of methods [3, 8]. There are limits to the conclusions that can be drawn from AE data but when it is relegated to simple frequency tables or analysed through inadequate practices, we do not make use of it in an optimal way [3]. The improvement of AE analysis has consistently received less attention than other methodological research areas [9]: recently, the CONSORT guidelines have been updated to include recommendations on how to properly collect and report harm outcomes [10], but guidance on the analysis of these outcomes remains very limited [2]. A better analysis of harm outcomes can lead to a clearer picture of the safety profile of an intervention earlier in its development, can allow patients and clinicians to make more informed treatment decisions and can improve the allocation of research resources.

Methods specifically designed to address the complexities of AE data could help achieve this - and many such methods already exist. A review conducted by Phillips et al. [11] found 73 methods designed specifically for the analysis of AEs in parallel two-arms phase II-IV RCTs. However, there is no evidence that these methods are being used in practice [11], due partly to lack of awareness about their existence and lack of guidance about when or how to implement them [12]. The evaluation of methods was identified as a key area for further research [6]: the present review contributes to this by identifying and describing suitable methods for AE analysis and by focusing on methods leveraging the hierarchical structures within the coding of the AEs to improve signal detection (the most common example of such a structure is the Medical Dictionary for Regulatory Activities (MedDRA), a standardised terminology to code medical occurrences used for safety monitoring and regulatory purposes [13, 14]). This review expands and advances the previous work conducted by Phillips et al. [11], which searched databases up to 2018, was broader in focus and did not conduct an in-depth review of methods leveraging the structure of AEs.

These methods propose an innovative strategy to address the challenges of AE analysis, by looking at meaningful groupings of events instead of treating each event individually and by exploiting known group structures in the data. This reflects approaches taken by regulators when considering the overall safety-risk profile of an intervention [5, 15–17]. It also takes advantage of the prevalent use of MedDRA coding, which has now been widely adopted as the standard for coding AEs [13, 18]. Such methods are promising candidates for improving AE analysis in RCTs.

A rapid preparatory review of the literature (unpublished) identified several new methods since the Phillips et al. review [11], which had identified six methods in that category. Therefore a new review of the methodological literature with a different, more refined focus was undertaken to identify and describe methods leveraging the structure of AE data. The scope of this review is limited to methods within the aforementioned category that can be used for the analysis of adverse events data from randomised controlled trials (data collected on emerging adverse events during a clinical trial and which include a randomised controlled arm) and does not include methods for the analysis of observational data or data from spontaneous reporting databases. The aims were to:

(i) identify sources describing novel statistical methods directly applicable to the analysis of AE data from two-arm parallel group RCTs and which used information on the relationships between different events, through the use of a hierarchical structure or grouping system,
(ii) summarise, categorise and map the key methodological characteristics of the methods,
(iii) summarise the relevant concepts, approaches taken by the methods and core considerations for users of the methods.

Through this review, we intended to create a comprehensive picture of the methodological landscape in the area, to sign-post relevant methods and implementation tools to applied statisticians, and to flag where further research is needed.

## Methods

### Study design and planning

This study is a review of published work on methodology. Methodological reviews of this kind are sometimes referred to as scoping reviews [19], following the framework described in [19, 20]. Our review includes both a search of different types of information sources and key aspects of systematic reviews, namely a transparent and reproducible process, a systematic search and a pre-registered protocol with clearly defined inclusion criteria and data charting procedures. The protocol for this study was pre-registered on Prospero (registration number CRD42023404082 [21]) and this review was written following the Preferred Reporting Items for Systematic reviews and Meta-Analyses extension for Scoping Reviews (PRISMA-ScR) guidelines [22].

### Eligibility criteria

Inclusion criteria:

(i) Present methods that could be used specifically for signal detection of potential adverse reactions using AE data from RCTs.
(ii) Peer-reviewed - publications in the form of a PhD were deemed eligible as they had to have undergone some review process as part of a successful defense, but if the method was also described in a journal article, this latter version was selected.

Exclusion criteria:

(i) Describe meta-analyses, data mining methods or methods requiring the use of data external to the trial (other than to inform priors), as they are not adequate for use in a final analysis of the data at the end of a trial.
(ii) Do not describe a method in enough detail for its implementation.
(iii) The method’s aim was not the detection of signals (e.g. the aim is subgroup analyses).

### Information sources and search strategy

We screened the Additional file 5 attached to Phillips et al. review [11] for methods fitting the eligibility criteria of the present review. The search strategy was based on the combination of three main concepts : “clinical trials”, “adverse events”/“signal detection” and “statistical models”, each of which was expanded through an iterative process into a list of possible synonyms and alternative phrasings, with feedback from a librarian. The new search was limited to the years 2018-2023, as it was assumed relevant sources before 2018 had been picked up by the Phillips et al. review search [11]. The search was validated by checking it could identify previously included sources and sources identified through different means.

The MEDLINE, Embase, Web of Science and Scopus databases (as recommended per the COCHRANE guidelines [23]) were searched in February 2023 and the databases were then monitored for new results matching the search strategy until April 2024. The search was conducted using English search terms only but no language restrictions were imposed. Accordingly, when “publication type” search filters were available, they were restricted to include only “Article” types (see Additional file 1 for the full search strategy). Bibliographies and reference lists of all sources included were also searched (see Additional file 2 for a summary of the information sources used).

### Selection process and data collection process

One reviewer (LdAN) screened each record and, in case of doubt, discussed it with a second reviewer (RP). The platform Rayyan [24] was used to remove duplicates and record eligibility decisions. The data were charted in LibreOffice Calc through the use of a customised data extraction form, piloted on a subset of eligible sources. Entries on the pilot form were checked by the second reviewer (RP) and disagreements were resolved through discussion. A final version of the data extraction form was agreed after discussion and the full data extraction process was completed by one reviewer (LdAN).

### Data items

Sources presenting novel statistical methods generally include a description of the method, a case study demonstrating practical implementation and simulations evaluating the performance of the method in different scenarios. Accordingly, where applicable, data were collected on **(i)** the source **(ii)** the methodology, **(iii)** the characteristics of the case study conducted, **(iv)** the characteristics of the simulation(s) conducted and **(v)** other comments/conclusions made by the authors. For the full data extraction template, see Additional file 3.

### Synthesis methods

Due to the methodological nature of the review, no systematic synthesis of results or statistical analysis such as those present in ’typical’ systematic reviews was relevant. The analyses conducted are primarily descriptive and were guided by the data extracted.

Each method was reviewed in detail to understand its overall structure and characteristics. Based on this, a categorization of the methods was used to present a simple overview of the analysis approaches used. Key methodological characteristics (data items best characterising the distinctive features of the methods, independent of the above categorization), were identified and each method was mapped onto these characteristics. The stated aims of the methods and their links with previously published methods were explored and illustrated through a narrative summary and an explanatory diagram.

Quality of source assessments and risk of bias assessments are not applicable to this review, as the units of analysis are statistical methods.

## Results

### Search results

**Fig. 1:**
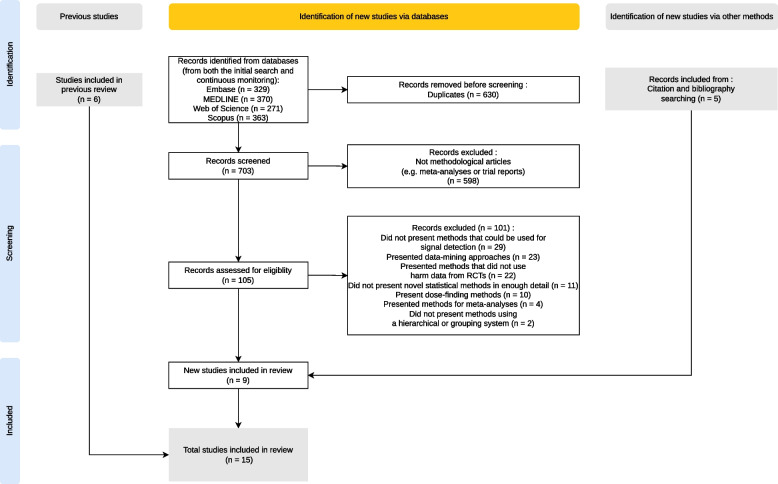
PRISMA flow diagram

The searches of the databases ran in February 2023 yielded a total of 1332 records. Subsequent monitoring of the databases for results matching the search strategy yielded 1 record. In total, five sources were identified through citation and bibliography searching, four sources through database searching and six sources through results from the Phillips et al. review [11]. A total of 15 sources of evidence were included [4, 5, 25–37] as detailed in the flow diagram in Fig. 1. The majority of records (85% of screened records) were excluded because they were not methodological articles (i.e. they were not articles that focused on presenting or discussing statistical methods). This high rate of exclusion was expected due to the difficult nature of running methodological searches.

Various descriptions of the same method in different sources, as well as slight variations of the same method (with no major methodological difference) were counted as one method and the version presented by the original authors was used as reference. One method identified was first presented in a PhD thesis [28] and later in a journal article [29]: only the journal article version of that method was used in the analysis, as it had undergone a more comprehensive peer-reviewing process and improvements by its authors. As a result, 14 sources are presented in the following analysis. These 14 sources described 18 different unique methods (see Table 5 in Additional file 4).

Out of the 14 sources included, 13 (presenting 13 methods) were journal articles and 1 [25] (presenting 5 methods) was a PhD thesis. Their dates of publication ranged from 2004 to 2023, with 6 sources published between 2019 and 2023. The number of citations of sources published between 2019 and 2023, which can be considered relatively new methods, ranged from 0 to 6, whilst the number of citations of sources published between 2004 and 2013 ranged from 6 to 195 (based on Google Scholar’s citation index).

### Categorisation of the methods and synthesis of the results

**Fig. 2:**
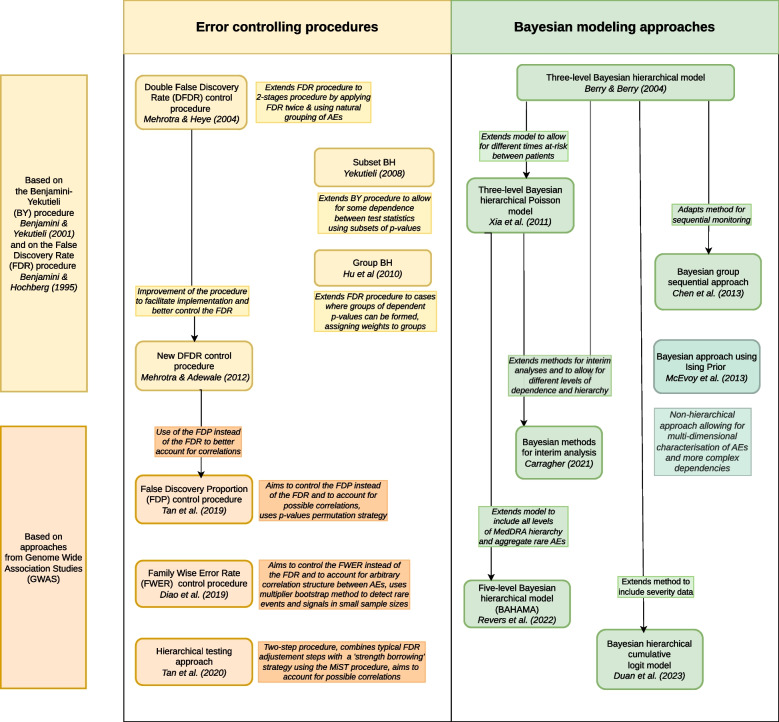
Diagram displaying the identified methods in chronological order with arrows describing how they relate to one another and textboxes giving a brief account of the methods’ particularities

We categorised methods into two groups, based on the general analysis approach employed: (1) Error controlling procedures, or alternatively methods conducting significance tests only, and (2) Bayesian modeling approaches, or alternatively methods producing treatment effect estimates. This follows the categorisation found in [25, 38]. The list of methods and their characteristics are presented in Tables 1 and 2. A summary of methodological characteristics by methods is presented in Table 3. The diagram in Fig. 2 presents a chronological overview of the methods and the links between them.

**Table 1:** Error controlling procedures for signal detection of potential adverse reactions using Adverse Events (AEs) data

Method ID	Source	Method	Error family	Type of outcomes	Nature of the data^a^	Timing of analysis	Restrictions on the events considered	Unique assignment of an AE to a group	Available software	Authors’ affiliations; funding sources (if disclosed)
M2	Mehrotra and Heyse (2004)	Double False Discovery Rate control (DFDR) procedure	False Discovery Rate control procedures	Binary	Summary data	Final	Tier 2 AEs^b^ only	Yes	R package c212 (partial implementation)^c^	Pharmaceutical Industry (Merck Research Laboratories)
M3	Yekutieli (2007)	Subset Benjamini-Hochberg (subset BH)	False Discovery Rate control procedures	Binary	Summary data	Final	None specified	No	R package c212^c^	Academia
M4	Hu et al. (2010)	Group Benjamini-Hochberg (group BH)	False Discovery Rate control procedures	Binary	Summary data	Final	None specified	Yes	R package c212^c^	Academia; funded by NSF and NIH grants^d^
M6	Mehrotra & Adewale (2012)	New DFDR control procedure	False Discovery Rate control procedures	Binary	Summary data	Final	Tier 2 AEs^b^ only	Unspecified	R package c212^c^	Pharmaceutical Industry (Merck Research Laboratories)
M9	Diao et al. (2019)	Family Wise Error rate control procedure	Family Wise Error rate control procedure	Binary	Patient-level data	Final	None specified	Unspecified	None available	Academia & Pharmaceutical Industry (Merck & Co)
M10	Tan et al. (2019)	False Discovery Proportion control procedure	False Discovery Proportion control procedure	Binary	Patient-level data	Final	None specified	Unspecified	None available	Academia & Pharmaceutical Industry (Merck & Co); funded by Merck & Co
M11	Tan et al. (2020)	Hierarchical testing approach	False Discovery Rate control procedures	Binary	Patient-level data	Final	None, all AEs reported included	Unspecified	R package MiST^e^(partial implementation)	Academia; funded by NSERC, authors supported by NCI, NSERC and NIH grants^f^

^a^Methods which use patient-level data are able to account for possible correlations within patients

^b^Based on the tiers framework for AE analysis, Tier 2 includes emerging AEs which are routinely collected during the trial, but do not make the object of a pre-specified hypothesis test. Tier 2 excludes rare spontaneous events which must be examined individually by experts

^c^[39]

^d^NSF: National Science Foundation (US), NIH: National Institutes of Health (US)

^e^[40, 41]

^f^NSERC: Natural Sciences and Engineering Research Council of Canada, NIH: National Institutes of Health (US), NCI: National Cancer Institute

**Table 2:** Bayesian modelling approaches for signal detection of potential adverse reactions using Adverse Events (AEs) data

Method ID	Source	Method	Type of outcomes	Nature of the data^a^	Timing of analysis	Restrictions on the events considered	Unique assignment of an AE to a group	Modeling structure	Available software	Authors’ affiliations; funding sources (if disclosed)
M1	Berry & Berry (2004)	Three-level hierarchical mixed model	Binary	Summary data	Final	Tier 2 AEs^b^ only	Yes	Hierarchical structure, 3 levels	R package c212^c^	Academia & Statistical Consulting Company (Berry Consultants)
M5	Xia et al. (2011)	Three-level hierarchical Poisson model	Binary, adjusted for time-at-risk	Summary data	Final	None, all AEs reported included	Yes	Hierarchical structure, 3 levels	No software, code used openly available	Academia & Pharmaceutical Industry (Amgen Inc.)
M7	Chen et al. (2013)	Bayesian group sequential approach	Binary	Summary data	Interim	None specified	Unspecified	Hierarchical structure, 3 levels	None available	Academia & Pharmaceutical Industry (Merck Serono)
M8	McEvoy et al.(2013)	Bayesian approach using Ising prior	Binary	Summary data	Final	All AEs reported can be included or only a few of clinical interest	No	Complex structure (graph structure)	No software, code used openly available	Academia & Drugs Regulator (US Food and Drug Administration)
M12-M14	Carragher (2021)	Bayesian hierarchical models for interim analyses (3 models)	Binary, adjusted for time-at-risk	Summary data	Interim	None specified	Unspecified	Hierarchical structure, 3 levels	R package c212^c^	Academia
M15-M16	Carragher (2021)	Bayesian hierarchical models for interim analyses (2 models)	Binary, adjusted for time-at-risk	Summary data	Interim	None specified	Unspecified	Hierarchical structure, 2 levels	R package c212^c^	Academia
M17	Revers et al. (2022)	Bayesian Hierarchical Model for the Detection of MedDRA-Coded Adverse Events (BAHAMA)	Counts	Summary data	Final	None, all AEs reported included	No	Hierarchical structure, 5 levels	R package BAHAMA^e^	Academia
M18	Duan et al. (2023)	Bayesian hierarchical cumulative logit model	Binary and ordinal	Summary data	Final	None specified	Unspecified	Hierarchical structure, 3 levels	None available	Academia & Pharmaceutical Industry (Merck & Co); supported by a National Cancer Institute Cancer Center grant

^a^Methods which use patient-level data are able to account for possible correlations within patients

^b^Based on the tiers framework for AE analysis, Tier 2 includes emerging AEs which are routinely collected during the trial, but do not make the object of a pre-specified hypothesis test. Tier 2 excludes rare spontaneous events which must be examined individually by experts

^c^[39]

^d^[40, 41]

^e^[34]

**Table 3:** Summary of methodological characteristics by methods

Characteristic	Classification		Methods
Type of outcomes	Binary		10 (M1, M2, M3, M4, M6, M7, M8, M9, M10, M11)
	Binary, adjusted for time-at-risk		6 (M5, M12-M16)
	Binary and ordinal		1 (M18)
	Counts		1 (M17)
Nature of the data	Patient-level data		3 (M9, M10, M11)
	Summary data		16^a^ (M1, M2, M3, M4, M5, M6, M7, M8, M10^a^, M12-M16, M17, M18)
Type of analysis	Interim analyses		6 (M7, M12-M16)
	Final analysis		12 (M1, M2, M3, M4, M5, M6, M8, M9, M10, M11, M17, M18)
Restriction on the events considered	Tier 2 AEs only		3 (M1, M2, M6)
	None specified, all AEs		15 (M3, M4, M5, M7, M8, M9, M10, M11, M12-M16, M17, M18)
	None specified, all AEs, with special considerations for rare AEs		2^a^ (M9^a^, M17^a^)
Structure of the Bayesian modeling approaches	Fixed hierarchical structure	2 levels	2 (M15, M16)
		3 levels	7 (M1, M5, M7, M12, M13, M14, M18)
		5 levels	1 (M17)
	Complex graph-like structure		1 (M8)
Unique assignment of an AE type to a group	Yes		4 ( M1, M2, M4, M5)
	No		3 (M3, M8, M17)
	Unspecified/unclear		11 (M6, M7, M9, M10, M11, M12-M16, M18)
Notion of error used in error controlling procedures	False Discovery Rate		5 (M2, M3, M4, M6, M11)
	False Discovery Proportion		1 (M10)
	Family Wise Error Rate		1 (M9)
Framework used	Frequentist		7 (M2, M3, M4, M6, M9, M10, M11)
	Bayesian		11 (M1, M5, M7, M8, M12-M16, M17, M18)
R package available for direct implementation	Yes		10 (M1, M3, M4, M6, M12-M16, M17)
	No		8 (M2, M5, M7, M8, M9, M10, M11, M18)
Authors’ affiliations	Academia only		9 (M3, M4, M11, M12-M16, M17)
	Academia and Drug Regulator		1 (M8)
	Academia and Statistical Consulting Company		1 (M1)
	Academia and Pharmaceutical Industry		5 (M5, M7, M9, M10, M18)
	Pharmaceutical Industry only		2 (M2, M6)

^a^A method is counted in more than one category

#### Error controlling procedures

Error controlling procedures work with *p*-values obtained from comparison tests between the intervention and the control arms for each AE (the choice of test is independent of the procedure). Typically, AEs are assigned to groups (e.g. by body systems) and *p*-values representative of these groups are computed (for instance, the smallest *p*-value of a group, i.e. its “strongest signal”). Adjustments specific to the procedure are performed on the *p*-values and AEs are flagged if and only if the adjusted *p*-values (typically both the *p*-value for the AE and the group representative *p*-value) obtained fall below a pre-specified threshold. In some cases, the choice of threshold is dependent on the procedure: for instance, there are constraints on the thresholds which ensure that the error is controlled at the desired level. The procedure ensures that some form of error is controlled at a pre-specified level.

Instead of aiming to control the type I error rate, three different notions of error, specific to multiple testing problems, are used : the Family Wise Error Rate (FWER), the False Discovery Rate (FDR) and the False Discovery Proportion (FDP) - see Table 4 for a summary of these notions. One of the key differences between the methods is the error rate they target : five methods (M2, M3, M4, M6, M11) use the FDR, one method uses the FDP (M10) and one method uses the FWER (M9).

**Table 4:** Notions of errors used in statistical methods for adverse events signal detection

Error notion	Summary
***Family Wise Error Rate (FWER):*** The FWER is the probability of making at least one type I error when testing a family of null hypotheses.	The FWER has long been used in multiplicity problems. It is most appropriate in scenarios where the presence of at least one type I error can compromise the validity of the conclusions drawn from testing the entire family of hypotheses - but can be overly conservative when this is not the case.^a^
***False Discovery Rate (FDR):*** A ’statistical discovery’ refers to a rejected null hypothesis. The FDR is the expected proportion of false discoveries among the set of all discoveries made (or the number of type I errors over the total number of rejected null hypotheses).	The FDR was developed in 1995 by Benjamini and Hochberg, as a more powerful control measure than the FWER in situations where we do not expect a small proportion of type I errors to invalidate the overall conclusions (e.g. when evaluating the overall safety profile of an intervention through several components).^a^
***False Discovery Proportion (FDP):*** The FDP is a random variable describing the proportion of false discoveries over all discoveries and whose expectation is the FDR.	The FDP is proposed by some authors as an alternative to the FDR. The argument used is that increased dependence between test statistics can lead to a skewed distribution of the FDP, in which case the FDR is a less meaningful measure.^b^

^a^[42]

^b^[35]

### Bayesian modelling approaches

Bayesian modeling approaches model AEs and their relationships with each other. They allow related events to *borrow strength* from each other: for instance, events with signals in the same group will lead to a stronger indication that there is a non-negligible treatment effect. Conversely, a lone signal in a group will ’lose strength’ - thus, these approaches also limit the identification of lone or ’outlier’ signals. Posterior estimates of treatment effects are obtained through Bayesian inference and AEs are flagged if the posterior probabilities obtained exceed a pre-specified threshold. The choice of threshold is independent of the approach chosen and different flagging rules can be applied to the same output from a model. For instance, [26] introduce a decision-theoretic approach to determine threshold values for flagging AEs. For each AE type, different weights can be assigned to the different types of signal misclassifications (e.g. one might choose to primarily minimise missed signals for severe AEs at the expense of a larger type I error rate).

For further details on the methods and their implementation, refer to the original papers.

### Key methodological characteristics

#### Type of outcomes

Ten methods (M1, M2, M3, M4, M6, M7, M8, M9, M10, M11) use only binary outcomes (yes/no occurrence of an AE in each participant for each AE type) with an underlying assumption that the time-at-risk is the same for all participants. They ignore the possible presence of recurrent events. In 5 of these methods (M2, M3, M4, M6, M11), other types of outcomes (such as continuous or ordinal) could theoretically be used, as long as a valid test statistic for between-arms comparison can be derived. We classify these methods as using binary outcomes as it is the default outcome in the sources.

One method (M18) considers binary outcomes and data on the severity of events, in the form of an ordinal variable with four levels. An AE type is flagged if either an imbalance in the incidence or in the severity level is detected.

Six methods (M5, M12-M16) allow for varying time-at-risk between participants but not for recurrent events, as they allow at most one occurrence of each AE type per participant to be counted. The reasons given for this in [25] are to avoid non-incidental AEs or a single participant being responsible for many AEs. Here the assumption must be made that the risk of an AE occurring is constant over time.

One method (M17) accounts for recurrent events but uses the total number of participants within an arm as an offset rather than the total time-at-risk.

#### Nature of the data

Fifteen methods (M1-M8, M12-M16, M17, M18) do not make use of patient-level data and so cannot account for correlations at the patient-level. They use summary data only, i.e. how many participants in each arm experienced each AE type (and when applicable, the total time at risk and/or total number of AE counts). Three methods (M9-M11) explicitly aim to account for possible correlations between AEs, either both within and across groups of AEs (M9) or only within groups (M10, M11), and require patient-level data. Note that an alternate way to construct summary test statistics using summary data only is proposed in (M10) and that possible negative correlations are considered in (M11).

#### Timing of analysis

The seven error controlling procedures are designed for final analyses of AE data. A distinctive feature of Bayesian approaches is the possibility to update the model with additional data at regular time intervals, such as for interim analyses: six Bayesian modeling approaches (M7, M12-M16) adapt existing methods for use in sequential monitoring. Three of these methods (M7, M12, M15) assume that the occurrences of events are independent. The other three methods (M13, M14, M16) consider possible dependencies of events across intervals, either at the higher level of the hierarchy considered (M13, M16) or at the lowest level (M14). The remaining five Bayesian approaches are designed for final analyses only.

In the case of interim analyses, the methods need to account for the new multiplicity problem that arises from conducting multiple comparisons in time - see [26] for a discussion on this.

#### Events considered

A tiered framework is proposed to categorise AEs [33]: Tier 1 includes all pre-specified events; Tier 2 includes emerging events, for which data is routinely collected but no analysis is pre-planned; and Tier 3 consists of “rare spontaneous reports of AEs” [33] which need to be examined by experts. Three methods (M1, M2, M6) explicitly restrict their analyses to Tier 2 AEs. Two of these methods (M2, M6) specify a first step in which extremely low-incidence events must be discarded. One method (M17) proposes a strategy of aggregating rare AEs at a higher level of the hierarchy instead of discarding them. Another method (M9) is described as allowing for the detection of rare events. One method (M8) can be applied to either all AEs or to a few of clinical interest only. Three methods (M5, M11, M17) explicitly mention considering all AEs reported. The remaining sources do not mention any restrictions on the AEs considered.

#### Hierarchical or group structure

All error controlling procedures (M2-M4, M6, M9, M10, M11) and nine Bayesian modelling approaches (M1, M5, M7, M12-M16, M18) require AE types to be grouped into one higher level. One method (M17) uses four levels of the MedDRA hierarchy. For the Bayesian hierarchical models, this gives rise to a two-level hierarchical model for two methods (M15, M16), to a three-level hierarchical model for seven methods ((M1, M5, M7, M12-M14, M18) and to a four-level hierarchical model for one method (M17). These modeling approaches are based on a vertical structure: an AE type either belongs to a group, in which case it has a relationship with all other AE types within that group, or it does not. One method (M8) allows for the use of a more flexible and complex modeling structure: any pair of two AE types can be defined as ’neighbours’ and the strength of the relationship (meaning how much information should be shared between neighbouring AEs) can be quantified through an interaction term which has a ’smoothing’ effect.

Note that methods use either ’body systems’ or ’system organ classes’ as the main type of group but that the two terms represent equivalent concepts and can be used interchangeably [18].

What does matter is how different methods exploit these structures, including how and how much information is exchanged between groups and how individual AEs are assigned to groups (the latter is independent of the method). Almost all methods use a pre-defined structure, but it is often understood that other assignments are possible as long as they are justifiable. Acceptable reasons cited to group AEs together vary slightly but are largely along the lines of : ’the groups must be based on clinically or biologically meaningful relationships’ or ’the groups must be constructed in a way that is relevant to the mechanism of action of the intervention/drug’. It is generally agreed that the assignment of AEs to groups must be established before starting the analysis and it seems that most methods work under the assumption that the assignment of an AE type to a group must be unique, although only four methods (M1, M2, M4, M5) say so explicitly. Exceptions to this are two methods (M3, M17) which are explicit that groups can be disjoint and one method (M8) that allows a flexible modeling approach.

### Other results

#### Treatment effect measures

While error controlling procedures directly conduct significance tests to flag AEs, the Bayesian modeling approaches provide estimates of treatment effects, which can be used as is or paired with a decision rule to flag AEs. The treatment effect measures used are the log odds ratio (M1, M7, M8, M18), log relative risk (M5, M8, M12-M16), risk difference (M8) and log rate ratio (M17).

#### Choice of threshold

Eight methods (M2, M5, M6, M12-M16, M18) justify the choice of threshold for flagging events based on data from simulations, one method (M8) proposes a two-stage flagging scheme with a rule to choose threshold values, one method (M10) requires pre-specification of one cut-off value and then relies on solving a constrained optimisation problem, and another method (M7) proposes a decision theoretic approach for choosing threshold values.

#### Software and implementation

WinBugs, BRugs, SAS JMP, R, JAGS and Stan were used by authors to implement methods. Only four sources included publicly available code (sources presenting methods M5, M8, M12-M16, M17), with varying degrees of reproducibility. We found two R packages allowing users to completely implement methods: the R package c212 [39] to fully implement methods M1, M3, M4 and M6, M12-M16 as well as the standard BH procedure [42], the Bonferroni correction and an unadjusted testing procedure and to partially implement method M2; and the R package BAHAMA [34] to implement method M17. The R package MiST [40, 41] allows users to implement the MiST testing procedure, part of the procedure used in method M11.

#### Authors’ affiliations and funding sources

Eleven out of 18 methods were developed either by authors with affiliations to universities only (9 methods: M3, M4, M11, M12-M16, M17), universities and a regulatory body (1 method: M8) or universities and a statistical consulting company (1 method: M1). 5 methods (M5, M7, M9, M10, M18) were developed by authors with affiliations to both universities and pharmaceutical companies, while only 2 methods (M2, M6) were developed by authors with affiliations to the pharmaceutical industry only. The affiliations of all authors named on each source were considered. While most sources (10 out 14) did not explicitly declare any sources of funding, 3 out of the 4 sources which did reported being supported by various national funding bodies. Only 1 source reported being funded by a pharmaceutical company, Merck & Co, which was also the company most represented in authors affiliations.

## Discussion

We identified a significant number of existing methods using a variety of analytical approaches. A third of the sources included were published from 2019. This indicates a continued development of methods in this area, along with newer methods building on older ones or exploiting methodological advancements from other fields (three methods originated from Genome-wide Association Studies (GWAS), where multiple testing problems are commonplace, albeit at different scales). Yet there is little evidence that these methods are making their way into practice, with the number of citations of recent methods remaining low. Based on the affiliations of the authors and the funding sources mentioned, regulatory bodies and the pharmaceutical industry do not seem to be the main drivers of method development in this area, although they are overall the main users of methods to detect signals for potential adverse reactions. While the International Council for Harmonisation of Technical Requirements for Pharmaceuticals for Human Use (ICH) played a major role in the development and uptake of the MedDRA for the coding of the AEs [18, 43], it is difficult to find mentions of specialised statistical methods for the analysis of AE data from clinical trials in ICH guidelines. Instead, the ICH guideline on Statistical Principles for Clinical Trials [44] emphasises the use of descriptive statistical methods, survival analysis methods, confidence intervals and graphical presentations. The guideline does advise researchers that they should take into account multiplicity issues and varying times-at-risk between participants, that the “risks associated with identified adverse effects should be appropriately quantified” [44] and that a “consistent methodology be used for the data collection and evaluation” [44], but it does not mention more sophisticated methods developed specifically for AE data analysis. We note that the designs of several of the methods identified in our review address the aforementioned recommendations. If methodological innovations in AE data analysis are largely led by academic researchers in statistics or biostatistics departments (which is the case for the methods considered in our review); and regulatory and pharmaceutical bodies are not actively contributing to or commissioning the development of more robust methods, this could in part explain the lack of use and general absence of discussion surrounding the value of such methods, along with other barriers such as the lack of readily available software to implement methods. Tangible changes in practice may require the involvement of all the groups who should be preoccupied with better signal detection of adverse reactions.

Eleven methods employed Bayesian approaches. Their role in safety assessment has been discussed in the literature: [45] argued that they could be a “more informative way of describing the potential risks [...] based on the accumulation of safety data” and [9] highlighted their value for continuous monitoring, straightforward probabilistic interpretations and predictions. We used a broad binary categorisation of methods in this review but some approaches aimed to combine different elements: Tan et al. [36] incorporate Bayesian ideas of strength borrowing under a frequentist approach and Hu et al. [30] present a Bayesian interpretation.

A third of the methods account for differential time-at-risk between participants, only one method makes use of severity data and only one method allows for recurrent events within the same participant. This is common in AE analysis [6], but means that the full extent of the information collected is not used. In five sources, authors note that extending the methods would be possible, either through the use of different test statistics or other regression models such as Poisson models.

Three methods explicitly restrict their analysis to Tier 2 AEs; some authors state that signal detection methods should not focus on rare events, as it is of limited value [4]. Nevertheless, one method (M9) specifically accounts for rare events, and other authors consider all reported AEs. Note that phase III trials are commonly not considered to be the place to detect such events [5] and we would instead expect to pick up signals for rarer events through post-marketing surveillance. The value of such methods is thus unclear and appraisal of these methods should include scenarios with rare events. Similarly, the value in practice of allowing for non-unique assignments of events to a group remains to be determined.

The choice of threshold to flag AEs is not always justified explicitly. Tan et al. [36] highlight that there is no consensus on how to choose scenario-specific values for thresholds/cut-offs and indeed, we observed a lack of standardised strategies to define or justify these.

All methods discussed are based on certain assumptions about how signals arise (for example that an existing treatment effect is more likely to affect events within the same body system), which might not be valid in every context and with which some investigators might disagree. Assumptions regarding possible relationships between AEs differ; the choice of method can reflect the user’s beliefs about underlying structures in the data.

### Limitations

Only one reviewer screened the search results and extracted the data - however, this is tempered by the fact that identifying a methods paper is quite clear-cut and the screening criteria did not lead to ambiguous cases. It is possible that some relevant sources were missed, if they were not accessible through the sources searched. There are limitations to using the number of citations as a proxy for method use. Lastly, we did not conduct a standardised quality appraisal of the studies, as there is a lack of tools to formally appraise methods papers.

A general limitation of the methods considered is that they are sensitive to the assignment of events to a certain group [4]. These methods rely on creating meaningful groupings of events - this is a broad notion, which can be interpreted and applied in a variety of ways, as identified in the results. It usually requires clinical input, can depend on the intervention evaluated, and relies on assumptions about an intervention’s mechanisms of actions, which can be hard to verify. The assignment of events to groups is ’step zero’ of the methods discussed; it creates an extra layer where possible issues might arise and which can influence the results obtained. When using these methods, the prior assignments of events to groups by clinicians or experts needs to be clear and justified.

There is little comparability possible between the simulations run across different sources, as the scenarios and performance measures considered often vary. To our knowledge, several of the methods (M7-M18) have only been appraised by the authors of the methods themselves, which creates a potential bias.

### Future research

Methodological scoping reviews are not adequate tools to conduct objective and critical appraisals of methods: an independent large scale simulation study (led by researchers with no conflicts of interest) would allow a clear comparison of the proposed methods; as well as applications to case studies to explore their performances in a range of real-world scenarios. This could inform guidelines on appropriate analytical approaches for AEs. Other gaps to be addressed include creating consensus on the best practices surrounding statistical methods for AE analysis; for example, addressing how suitable thresholds for flagging events should be determined. Finally, further work could be conducted to extend existing methods to make use of other types of data and include covariates or patient-level data in the models.

## Conclusions

This work is the first methodological review of methods leveraging the structure of AE data for signal detection in clinical trials. We identified 14 sources describing 18 novel statistical methods, highlighting the non-negligible number of existing methods for signal detection in clinical trials, the wide variety in methodological approaches and the continued methodological evolution in this area (10 of the methods identified were published in the last 5 years). While this is promising, it also means that trial statisticians are faced with a large number of very different approaches, with no indication on which to choose. These developments must go hand in hand with objective appraisal and guidance, otherwise they will not be matched by improvements in practice.

## Supplementary Information

Additional file 1: Search strategy. The document AdditionalFile1.odt contains the full search strategy used for each database. The PRISMA-S guidelines [46] were followed to report the search strategy.

Additional file 2: Information Sources. The document AdditionalFile2.odt contains the list of all sources searched, the dates of the searches and the interfaces used.

Additional file 3: Data extraction template. The document AdditionalFile3.odt contains the full data extraction template used.

Additional file 4: List of all methods. The document AdditionalFile4revised.pdf contains the list of all statistical methods included in the review.

## Data Availability

No datasets were generated or analysed during the current study.

## Abbreviations

AEs: Adverse events
FDP: False discovery proportion
FDR: False discovery rate
FWER: Family wise error rate
GWAS: Genomewide association studies
ICH: International Conference on Harmonisation
MedDRA: Medical dictionary for regulatory activities
PRISMA: Preferred reporting items for systematic reviews and meta-analyses
RCTs: Randomised controlled trials
