# Supplementary material for: Statistical methods leveraging the hierarchical structure of adverse events for signal detection in clinical trials: a scoping review of the methodological literature
Source: BMC Med Res Methodol. 2024 Oct 28;24:253. doi: 10.1186/s12874-024-02369-1 (PMC11514772; doi:10.1186/s12874-024-02369-1)
Supplement: Supplementary file 4 — Additional file 4: List of all methods. The document AdditionalFile4revised.pdf contains the list of all statistical methods included in the review. [file 12874_2024_2369_MOESM4_ESM.pdf]

Table 5 List of all statistical methods included in the review

Method ID	Method	Author(s)	Publication year
M1	Bayesian three-level hierarchical mixed model	S. Berry, D. Berry	2004
M2	Double False Discovery Rate (DFDR) control procedure	D. Mehrotra, J. Heyse	2004
M3	False Discovery Rate (FDR) control for non-positively regression dependent test statistics (known as subset BH[1])	D. Yekutieli	2008
M4	Group False Discovery Rate (FDR) control procedure (known as group BH[1])	J. Hu, H. Zhao, H. Zhou	2010
M5	Bayesian three-level hierarchical Poisson model	H. Xia, H.Ma, B. Carlin	2011
M6	New Double False Discovery Rate (DFDR) control procedure	D. Mehrotra, A. Adewale	2012
M7	Bayesian three-level hierarchical model for multiple interim analyses	W. Chen, N. Zhao, G. Qin, J. Chen	2013
M8	Bayesian hypothesis testing approach	B. McEvoy, R. Nandy, R. Tiwari	2013
M9	Multiplier bootstrap method for controlling the family wise error rate	G. Diao, G Liu, D. Zeng, W. Wang, X. Tan, J. Heyse, J. Ibrahim	2019
M10	Two-stage False Discovery Proportion (FDP) control procedure	X. Tan, G. Liu, D. Zeng, W. Wang, G. Diao, J. Heyse, J. Ibrahim	2019
M11	Hierarchical testing approach	X. Tan, B. Chen, J. Sun, T. Patel, J. Ibrahim	2020
M12	Bayesian three-level hierarchical Poisson model for interim analyses of independent intervals	R. Carragher	2021
M13	Bayesian three-level hierarchical Poisson model for interim analyses of intervals with some dependence at the higher structure level	R. Carragher	2021
M14	Bayesian three-level hierarchical Poisson model for interim analyses of intervals with some dependence at the lower structure level	R. Carragher	2021
M15	Bayesian two-level hierarchical Poisson model for interim analyses of independent intervals	R. Carragher	2021
M16	Bayesian two-level hierarchical Poisson model for interim analyses of intervals with some dependence at the higher structure level	R. Carragher	2021
M17	BAHAMA : multi-stage Bayesian hierarchical Poisson model	A. Revers, M. Hof, A. Zwinderman	2022
M18	Bayesian hierarchical cumulative logit model	J. Duan, B. Gajewski, P. Sen, J. Wick	2023

[1]BH stands for the Benjamini-Hochberg procedure
