# Supplementary material for: Statistical methods leveraging the hierarchical structure of adverse events for signal detection in clinical trials: a scoping review of the methodological literature
Source: BMC Med Res Methodol. 2024 Oct 28;24:253. doi: 10.1186/s12874-024-02369-1 (PMC11514772; doi:10.1186/s12874-024-02369-1)
Supplement: Supplementary file 5 [file 12874_2024_2369_MOESM1_ESM.odt]

Additional File 1 - Search Terms by Database

| Database: Embase |
| --- |
| Interface used : Embase (Embase.com) |
| Date of the search: 20/02/2023 |
| Sources: Embase, Medline, Embase classic, Pubmed-not-Medline |
| Years covered by the search: 2018-2023 |
| Limits applied: Only title and abstract searched (:ti,ab) |
| Language restriction : None |
| Publication types included : Articles, Articles in Press |
| Other filters: None |
| Search | Search term | Number of results |  |
| 1 | ('clinical stud*' OR 'clinical trial*' OR 'clinical trial (topic)' OR 'randomi?ed control* trial*' OR 'randomized controlled trial (topic)'):ti,ab | 169826 |  |
| 2 | ('pharmacovigilance' OR 'drug monitoring' ):ti,ab OR ( ('adverse drug event*' OR 'adverse drug reaction*' OR  'adverse event*' OR 'adverse reaction*' OR 'side effect*' OR 'drug reaction*') NEAR/5 ('monitor*' OR 'analys*' OR 'flag*' OR 'signal*' OR 'detect*' OR 'identif*' OR 'evaluat*' OR 'screen*' OR 'assess*' OR 'approach*' OR 'extract*') ):ti,ab OR (('monitor*' OR 'analys*' OR 'flag*' OR 'signal*' OR 'detect*' OR 'identif*' OR 'evaluat*' OR 'screen*' OR 'assess*' OR 'approach*' OR 'extract*') NEAR/3 ('adverse' OR 'undesirable' OR 'harm*' OR 'serious' OR 'toxic*' OR 'complicat*' OR 'noxious') NEAR/3 ('effect*' OR 'reaction*' OR 'event*' OR 'outcome*' OR 'experience*')) | 22130 |  |
| 3 | ('cluster*' OR 'nested' OR 'bayes*' OR 'likelihood ratio' OR 'markov' OR 'monte carlo' OR 'hierarch*' OR 'hierarchical model*' OR 'multi*level*' OR 'mixed*effect*' OR 'random*effect*' OR 'mixture*model*' OR 'medical dictionar*' OR 'meddra' OR 'preferred terms' OR 'system organ class' OR 'body system*' OR 'signal detection' OR 'false discovery'):ti,ab | 203015 |  |
| 4 | #1 AND #2 AND #3 | 328 |  |

| Intermediary searches |
| --- |
| Final search |

| Database: MEDLINE |
| --- |
| Interface used : [Ovid](http://ezproxy.library.qmul.ac.uk/login?url=http://ovidsp.ovid.com/ovidweb.cgi?T=JS&PAGE=dblist) |
| Date of the search: 20/02/2023 |
| Resources:  Ovid MEDLINE(R) and Journals@Ovid Full Text |
| Publication Year: 2018-Current |
| Limits applied: Only abstract and title searched (.tw.) |
| Language restriction : None |
| Other filters: None |
| Search | Search term | Number of results |  |
| 1 | ("clinical stud*" or "clinical trial*" or "randomi?ed control* trial*").tw. or exp Clinical Trials as Topic/ | 253222 |  |
| 2 | ("pharmacovigilance" or "drug monitoring").tw or ( ("adverse drug event*" or "adverse drug reaction*"  or "adverse event*" or "adverse reaction*" or "side effect*" or "drug reaction*") adj6 ("monitor*" or "analys*" or "flag*" or "signal*" or "detect*" or "identif*" or "evaluat*" or "screen*" or "assess*" or "approach*" or "extract*") ).tw or (("monitor*" or "analys*" or "flag*" or "signal*" or "detect*" or "identif*" or "evaluat*" or "screen*" or "assess*" or "approach*" or "extract*") adj4 ("adverse" or "undesirable" or "harm*" or "serious" or "toxic*" or "complicat*" or "noxious") adj4 ("effect*" or "reaction*" or "event*" or "outcome*" or "experience*")).tw. or exp "Drug-Related Side Effects and Adverse Reactions"/ | 50047 |  |
| 3 | ("cluster*" or "nested" or "bayes*" or "likelihood ratio" or "markov" or "monte carlo" or "hierarch*" or "hierarchical model*" or "multi*level*" or "mixed*effect*" or "random*effect*" or "mixture model*" or "medical dictionar*" or "meddra" or "preferred terms" or "system organ class" or "body system*" or "signal detection" or "false discovery").tw. | 484656 |  |
| 4 | 1 and 2 and 3 | 370 |  |

| Intermediary searches |
| --- |
| Final search |

| Database: Web of Science |
| --- |
| Interface :  Web of Science |
| Date of the search: 20/02/2023 |
| Sources: Web of Science |
| Years covered by the search: 2018-current |
| Limits applied: Only title, abstract, author keywords, and Keywords Plus searched (TS=) |
| Language restriction : None |
| Publication types included : Early Access, Open Access, Enriched Cited References (Review Articles excluded) |
| Other filters: Document type restricted to Article or Early Access |
| Search | Search term | Number of results |
| 1 | (  (TS=("clinical trial*" OR "clinical stud*" OR "randomi?ed control* trial*") ) AND ( (TS = ("pharmacovigilance" OR "drug monitoring") ) OR (TS=( ("adverse drug event*" OR "adverse drug reaction*"  OR "adverse event*" OR "adverse reaction*" OR "side effect*" OR "drug reaction*") NEAR/5 ("monitor*" OR "analys*" OR "flag*" OR "signal*" OR "detect*" OR "identif*" OR "evaluat*" OR "screen*" OR "assess*" OR "approach*" OR "extract*")  ) ) OR ( TS = (    ("monitor*" OR "analys*" OR "flag*" OR "signal*" OR "detect*" OR "identif*" OR "evaluat*" OR "screen*" OR "assess*" OR "approach*" OR "extract*") NEAR/3 ("adverse" OR "undesirable" OR "harm*" OR "serious" OR "toxic*" OR "complicat*" OR "noxious") NEAR/3 ("effect*" OR "reaction*" OR "event*" OR "outcome*" OR "experience*")  ) )) AND (TS= ("cluster*" OR "nested" OR "bayes*" OR "likelihood ratio" OR "markov" OR "monte carlo" OR "hierarch*" OR "hierarchical model*" OR "multi*level*" OR "mixed*effect*" OR "random*effect*" OR "mixture model*" OR "medical dictionar*" OR "meddra" OR "preferred terms" OR "system organ class" OR "body system*" OR "signal detection" OR "false discovery"))) | 271 |

| Database: Scopus |
| --- |
| Interface : Scopus |
| Date of the search: 20/02/2023 |
| Sources: Scopus |
| Years covered by the search: 2018-current |
| Limits applied: Only title, abstract and keywords searched (TITLE-ABS-KEY) |
| Language restriction : None |
| Publication types included : Article |
| Other filters: None |
| Search | Search term | Number of results |  |
| 1 | ( ( TITLE-ABS("clinical trial*" OR "clinical stud*" OR "randomi?ed control* trial*") )  AND  ( TITLE-ABS-KEY("pharmacovigilance" OR "drug monitoring") OR (TITLE-ABS-KEY("adverse drug event*" OR "adverse drug reaction*" OR "adverse event*" OR "adverse reaction*" OR "side effect*" OR "drug reaction*") W/5 TITLE-ABS-KEY("monitor*" OR "analys*" OR "flag*" OR "signal*" OR "det" OR "identif*" OR "evaluat*" OR "screen*" OR "assess*" OR "approach*" OR "extract*") ) OR ( TITLE-ABS-KEY("monitor*" OR "analys*" OR "flag*" OR "signal*" OR "det" OR "identif*" OR "evaluat*" OR "screen*" OR "assess*" OR "approach*" OR "extract*") W/3 ("adverse" OR "undesirable" OR "harm*" OR "serious" OR "toxic*" OR "complicat*" OR "noxious") W/3 ("effect*" OR "reaction*" OR "event*" OR "outcome*" OR "experience*") ) ) AND (TITLE-ABS-KEY("cluster*" OR "nested" OR "bayes*" OR "likelihood ratio" OR "markov" OR "monte carlo" OR "hierarch*" OR "hierarchical model*" OR "multi*level*" OR "mixed*effect*" OR "random*effect*" OR "mixture model*" OR "medical dictionar*" OR "meddra" OR "preferred terms" OR "system organ class" OR "body system*" OR "signal detection" OR "false discovery")) ) AND PUBYEAR > 2017 | 363 |  |
