# Supplementary material for: Statistical methods leveraging the hierarchical structure of adverse events for signal detection in clinical trials: a scoping review of the methodological literature
Source: BMC Med Res Methodol. 2024 Oct 28;24:253. doi: 10.1186/s12874-024-02369-1 (PMC11514772; doi:10.1186/s12874-024-02369-1)
Supplement: Supplementary file 6 [file 12874_2024_2369_MOESM2_ESM.odt]

Additional File 2 - Information Sources

| Source name | Date searched | Interface used |
| --- | --- | --- |
| MEDLINE (database) | February 2023¹ | Ovid |
| Embase (database) | February 2023¹ | Embase |
| Web of Science (database) | February 2023¹ | Web of Science |
| Scopus (database) | February 2023¹ | Scopus |
| Studies included in previous search by Phillips et al.² | February 2023 | Studies listed in Additional File 5² |
| Bibliographies of included studies | February 2023 | Various³ |
| References cited in included studies | February 2023 | Various⁴ |

¹ The searches were subsequently monitored via email alerts.

² Phillips, R., Sauzet, O. & Cornelius, V. Statistical methods for the analysis of adverse event data in randomised controlled trials: a scoping review and taxonomy. BMC Med Res Methodol 20, 288 (2020). https://doi.org/10.1186/s12874-020-01167-9

³ The bibliography was checked through the publication platform. 

⁴ When available, the reference list was checked through the publication platform. Otherwise, Google Scholar was used.
