# Supplementary material for: Statistical methods leveraging the hierarchical structure of adverse events for signal detection in clinical trials: a scoping review of the methodological literature
Source: BMC Med Res Methodol. 2024 Oct 28;24:253. doi: 10.1186/s12874-024-02369-1 (PMC11514772; doi:10.1186/s12874-024-02369-1)
Supplement: Supplementary file 7 [file 12874_2024_2369_MOESM3_ESM.odt]

Additional File 3 - Data Extraction Template

Abbreviations

AEs - Adverse Events

RCT - Randomised Controlled Trial

Template 

| For each study included in the review |
| --- |
| Category | Data item | Response type |
| Study characteristics | 1. Study ID | Numerical |
| 2. Method ID | Numerical |
| 3. Title | Free text |
| 4. Author(s) | Free text |
| 5. Author(s) affiliation | Free text |
| 6. Research funding body | Free text |
| 7. Year of publication | Numerical (Year) |

| For each method presented in a study included in the review |
| --- |
| Category | Subcategory | Data item | Response type |
| Methodological characteristics |  | 8. Name and/or brief description of the method | Free text |
| Characteristics of the AE data considered | 9. Any restriction(s) on the type of AEs considered | Free text |
| 10. Information the AE data must include | Free text |
| 11. Is the time-at-risk assumed to be the same for all patients ? | Free text |
| 12. What are the assumptions made on dependencies between the AEs ? | Free text |
| 13. If applicable, what are the assumptions made on dependencies between the test statistics used ? | Free text |
| 14. How are AEs assigned to a group ? | Free text |
| 15. Is that assignement unique ? | Yes/No/Not mentioned |
| Subgroup characteristics | 16. Nature of the data | Patient-level data / Summary data |
| 17. Type of trial considered | Two-arm RCT / Other |
| 18. Timing of the analysis | Final analysis / Sequential monitoring |
| 19. Type of events | Emerging events / Pre-specified events |
| 20. Approach used | Frequentist approach / Bayesian approach |
| 21. Type of data | Binary / Counts / Ordinal |
| 22. Type of author affiliation | Academia / Industry / Academia and industry |
| Model characteristics | 23. Hierarchy/grouping system used | Free text |
| 24. Model | Free text |
| 25. Prior (if applicable) | Free text |
| 26. Number of stages or levels | Numerical |
| 27. Other model details | Free text |
| 28. Description of the signal detection strategy | Free text |
| 29. Measure of treatment effect used | Free text |
| 30. Proposed flagging threshold and threshold choice strategy (if applicable) | Free text |
| 31. Assumptions made by the model with regards to the hierarchy/grouping system | Free text |
|  | 32. If applicable, on which existing model or previous study does the method build on ? | Free text |
| 33. If applicable, what is the change or extension proposed by the method | Free text |
| Implementation characteristics |  | 34. Software used to implement the method | Free text |
| 35. Is the code available ? If yes, where ? | Yes/No, Free text |
|  | 36. Has software been developed specifically for the implementation of the method ? If yes, what ? | Yes/No, Free text |
| Simulation study characteristics |  | 37. Was the model evaluated through simulations ? | Yes/No |
| 38. Description of scenarios considered in the simulation study (if applicable) | Free text |
| 39. Type of trial considered in the simulation study (if applicable) | Free text |
| 40. Description of the comparisons undertook with other models in the simulation study (if applicable) | Free text |
| 41. Performance measure(s) used in the simulation study (if applicable) | Free text |
| 42. Conclusions of the simulation study (if applicable) | Free text |
| 43. Sensitivity analyses conducted in the simulation study (if applicable) | Free text |
| Case study characteristics |  | 44. Case study on which the model was tested (if applicable) | Free text |
| 45. Description of the comparaisons undertook with other models in the case study (if applicable) | Free text |
| 46. Performance measure(s) used in the case study (if applicable) | Free text |
| 47. Conclusion(s) of the case study (if applicable) | Free text |
| Other information included in the study |  | Other recommendations/conclusions made by the authors | Free text |
|  | According to the authors, setting(s) in which the model works best | Free text |
|  | Possibility to extend the model to include other types of data | Free text |
|  | Limitations described by the authors | Free text |
|  | Other comments | Free text |

If information on a data item was missing from a source, it was recorded as "Not mentioned".

If a data item was not applicable to a source, it was recorded as "Not applicable".

Due to the nature of the review, missing items did not have a large impact on the synthesis of results.
